# Individual Contributions of Adverse Childhood Experiences to Adolescent Substance Use

**DOI:** 10.1080/10826084.2026.2679709

**Published:** 2026-06-05

**Authors:** Chelsea R. Moore, Philip T. Veliz, Bingxin Chen, Chaewon Lim, Sarah A. Stoddard

**Affiliations:** a School of Nursing, University of Michigan, Ann Arbor, Michigan, USA; b Division of General Pediatrics and Adolescent Health, Department of Pediatrics, University of Minnesota Medical School, Minneapolis, Minnesota, USA; c Health Behavior and Health Equity, University of Michigan, Ann Arbor, Michigan, USA

**Keywords:** Adverse childhood experiences, adolescent, tobacco use, marijuana use, alcohol use

## Abstract

**Background:**

Adverse childhood experiences (ACEs) are associated with adolescent substance use. However, the influence of individual ACEs after controlling for concurrent ACEs is understudied.

**Methods:**

Using longitudinal data from the Future of Families and Child Wellbeing Study (*n* = 3200), we evaluated binary logistic models to test associations from 11 individual ACEs at ages 3–9 (physical abuse; emotional abuse; neglect; housing instability; food insecurity; community violence; parental depression, problematic substance use, intimate partner violence [IPV], incarceration, and death) to adolescent cigarette, marijuana and heavy alcohol use at age 15, while controlling for sociodemographic characteristics and co-occurring ACEs.

**Results:**

Marijuana use was associated with neglect (aOR = 1.67, 95% CI = 1.08, 2.61) and parental mental illness (aOR = 1.28, 95% CI = 1.01, 1.62). Heavy alcohol use was associated with emotional abuse (aOR = 2.59, 95% CI = 1.23, 5.48). No ACEs were individually associated with cigarette use.

**Conclusions:**

Findings suggest a narrow set of ACEs may be driving well-documented associations between composite ACE indices and substance use outcomes. Emotional abuse may be a singularly salient risk factor for adolescent heavy alcohol use. Neglect and parental mental illness may be stronger risk factors for adolescent marijuana use. Findings provide guidance for tailored interventions and suggest composite ACE indices may incorrectly estimate adolescents’ risk for substance use.

## Introduction

Adolescent substance use, including marijuana, cigarette, and heavy alcohol use, is a major public health concern because of its profound risks for youth health and development. Recent surveys of United States (U.S.) high school students found approximately 17% reported past-month marijuana use, 4% reported past-month cigarette use, and 22% reported past-month alcohol use, with 9% binge-drinking (Centers for Disease Control and Prevention [CDC], 2026). Adolescent substance use has been associated with adult problematic substance use/substance use disorder, poorer mental and physical health (e.g., depression, neurocognitive changes, and illness), and legal and social problems (e.g., poorer academic performance and impaired relationships), which can have cascading consequences throughout life (Hall et al., 2016; Steinfeld & Torregrossa, 2023).

Adverse childhood experiences (ACEs), potentially traumatic circumstances that occur before age 18, are a well-established risk factor for adolescent/young adult substance use (Azagba et al., 2025; Hoffmann & Jones, 2022; Rodrigues et al., 2025; Rogers et al., 2022). Initially, researchers conceptualized ACEs as including abuse (physical, emotional/verbal, and sexual); neglect (physical and emotional); and other significant household stressors, like living with someone with mental illness or problematic substance use, living with someone who has been incarcerated, or having caregivers experience intimate partner violence (IPV) or divorce/separation (Felitti et al., 1998). More recently, the ACEs concept has been expanded to include additional adversities that similarly negatively affect health and development, like financial hardship, discrimination, and community violence (SmithBattle et al., 2022).

ACEs are commonly measured as a composite index of the number of different ACE types an individual ever experienced (Felitti et al., 1998). Exposure to more ACEs has been associated with a range of poor health and wellbeing outcomes, including adolescent/young adult alcohol, tobacco, and marijuana use, and substance use disorders (Azagba et al., 2025; Hoffmann & Jones, 2022; Rodrigues et al., 2025; Rogers et al., 2022). Moreover, ACEs are prevalent – estimates suggest 17% of U.S. adults experienced four or more ACEs (CDC, 2024a, 2024b).

Existing literature highlights the significant role that accumulating ACEs play in adolescent substance use; yet, identifying the most detrimental individual (or subsets of) ACEs may be critical for developing tailored strategies to reduce substance use among adolescents at highest risk. Further, emerging literature suggests distinct ACEs may influence outcomes *via* unique mechanisms, which may be overlooked when analyzing composite ACE indices, with implications for intervention development (Qeadan et al., 2025). While substantial literature has documented associations between individual ACEs *separately* (i.e., not accounting for co-occurring ACEs) and substance use outcomes (Rogers et al., 2022), individual ACEs analyzed within the context of other ACEs have been understudied (Qeadan et al., 2025; Rogers et al., 2022). This is problematic because evidence indicates ACEs are interrelated (Dong et al., 2004). Thus, identifying the effects of a single ACE without considering correlated ACEs may lead to biased estimates.

Of the limited existing studies investigating ACE effects after accounting for co-occurring ACEs, most have evaluated adult outcomes (Qeadan et al., 2025; Rogers et al., 2022). Studies on adolescent substance use report mixed findings (Afifi et al., 2020; Karamanos et al., 2022). Afifi et al. (2020) reported most of the twelve ACEs included in their study were associated with substance use outcomes, including cigarette, e-cigarette, alcohol, and marijuana use, in a sample of Canadian adolescents. Alternatively, Karamanos et al. (2022) found a smaller set of ACEs were associated with drug use in United Kingdom adolescents. Additional studies are needed to clarify the currently mixed evidence. Thus, this study explores the association between 11 individual ACEs and adolescent marijuana, cigarette, and heavy alcohol use, in a U.S.-based sample using simultaneous modeling to account for the interrelatedness of ACEs.

## Methods

### Sample

Data come from the Future of Families and Child Wellbeing Study (FFCWS, 2024), a cohort study of children born in 20 large U.S. cities between 1998 and 2000. The FFCWS oversampled unmarried mothers, resulting in a large proportion of participants with minoritized ethnoracial identities and low household incomes. At baseline, mothers were surveyed in the hospital at their child’s birth, with fathers surveyed in the hospital or *via* telephone when possible. Follow-up surveys were administered (predominantly *via* telephone, but occasionally in person; see (FFCWS, 2024) for additional details) with the youth’s caregivers (predominantly mothers and/or fathers, hereafter called *parents*) when the youth was about 1, 3, 5, 9, and 15 years old. Youth were interviewed at ages 9 and 15 (FFCWS, 2024). At age 15, about 73% of the baseline sample remained. Additional information on the FFCWS is available elsewhere (Reichman et al., 2001).

Our analytic sample (*n* = 3200; 65% of the baseline sample) consists of youth with data on at least one substance use outcome at age 15; whose primary caregiver at ages 1–9 was their biological mother or father; and whose primary caregiver completed at least one in-home survey at ages 3, 5, or 9 (where several ACEs were measured). Parent participation in the in-home surveys at ages 3, 5, and 9 were 79%, 81%, and 77%, respectively (FFCWS, 2024). Only 13 adolescents were omitted from the sample for missing substance use data after accounting for the other eligibility criteria.

### Measures

#### Substance use

All substance use variables, including cigarette, marijuana, and heavy alcohol use, were youth-reported at age 15 and were dichotomized to represent the presence (1) or absence (0) of each outcome. Cigarette use captured whether the adolescent smoked cigarettes at least once in the past month. Heavy alcohol use (i.e., binge drinking) captured whether the adolescent drank five or more alcoholic beverages in a day at least once within the past year. Marijuana use captured whether the adolescent used marijuana at least once in the past year.

#### Adverse childhood experiences (ACEs)

Eleven ACEs were parent-reported. Aligned with substantial existing literature on ACEs, ACEs were dichotomized to indicate the presence (1) or absence (0) of each ACE at any point in the first 9 years of the youth’s life (measured at ages 1, 3, 5, and/or 9, with some variation across ACEs).

Physical abuse was measured with items from the Parent-Child Conflict Tactics Scale (CTS) Physical Assault subscale (Straus et al., 1998) at ages 3, 5, and 9. Items assessed whether the child was hit on the bottom with a belt/hard object, spanked on the bottom with a bare hand, slapped, shook, or pinched. A child was indicated as experiencing physical abuse if they experienced any item six or more times in a year. This conservative threshold discounts any “one off” experiences and aligns with existing ACE research using FFCWS data (Gajos et al., 2023; Moore et al., 2025). (Although we emphasize no violence toward a child is considered safe.) Other CTS variables, described next, follow this cutoff for dichotomization for the same reasons. Cronbach’s alpha was 0.92.

Emotional abuse was measured with items from the CTS Psychological Aggression subscale (Straus et al., 1998) at ages 3, 5, and 9, with items asking how frequently a caregiver threatened to kick the child out of the house or physically harm the child; called the child dumb/lazy; or shouted, yelled, screamed, or swore at the child. A child was indicated as experiencing emotional abuse if they experienced any item six or more times in a year. Cronbach’s alpha was 0.92.

Neglect was measured with items from the CTS Neglect subscale (Straus et al., 1998) at ages 3, 5, and 9, with items asking how frequently the child was not taken to the doctor when necessary; was left home alone; or a parent was unable to show affection, hug, or tell the child they were loved/appreciated. Neglect was indicated if a child experienced any item six or more times in a year. Cronbach’s alpha was 0.87.

Parental incarceration was indicated if the mother or father spent time in jail at ages 1, 3, 5, or 9.

Parental problematic substance use, measured at ages 1, 3, 5, and 9, was indicated if the mother or father sought professional help for alcohol/drug use, self-reported alcohol/drug use interfered with daily functioning, or met criteria for probable substance dependence disorder based on the Composite International Diagnostic Interview – Short Form (CIDI-SF; Kessler et al., 1998; age 3 only).

Parental mental illness, measured at ages 1, 3, 5, and 9, was indicated if the mother or father reported treatment for depression or anxiety by a mental health professional or if they met conservative criteria for probable depression/anxiety using the CIDI-SF (Kessler et al., 1998).

Parental IPV, measured at ages 1, 3, 5, and 9, was indicated if the mother or father experienced physical, psychological, or sexual violence perpetrated by any partner “often” (*vs.* “sometimes” or “never”), *and* if the child spent some time with the victimized parent. Example IPV items included being hit with an object, slapped or kicked; being criticized or insulted; or being made to do unwanted sexual things. Cronbach’s alpha was 0.98.

Parental death was indicated if the child’s biological mother or father died before age 9.

Housing instability, measured at ages 1, 3, 5, and 9, was indicated if the mother or father reported they did not pay their rent/mortgage in full, were evicted for not paying their rent/mortgage, moved in with other people due to financial problems, or stayed in a place not meant for regular housing (e.g., a car, abandoned building, or shelter). Cronbach’s alpha was 0.70.

Food insecurity was measured at ages 3 and 5 with child-specific questions from the U.S. Department of Agriculture Food Security Module. Primary caregivers reported if (yes/no) or how frequently they could not feed the child balanced meals or relied on few kinds of low-cost food to feed the child; the child was not eating enough; they had to cut the size of the child’s meals; the child skipped meals or did not eat for an entire day; or the child was hungry, but the caregiver could not afford more food. For questions assessing frequency, responses included “never” (coded as 0), “sometimes true” (1), or “often true” (2). Questions specifically assessing how often children skipped a meal because of money were coded as “never” (0), “only 1 or 2 months” (1), “some months but not every month” (2), or “almost every month” (3). Aligned with FFCWS guidelines, scores were summed; children with scores >2 were indicated as experiencing food insecurity (coded as 1) *vs.* food security (0). Cronbach’s alpha was 0.79.

Community violence exposure, measured at ages 3, 5, and 9, included items assessing how often caregivers saw someone get hit, slapped, punched or beaten up; threatened/attacked with a weapon; shot at; or killed because of violence (Selner-O’Hagan et al., 1998). Respondents also reported if they personally experienced such violence. To discount any “one off” experiences of violence and better capture persistent violence in the environment, we summed the number of times a respondent reported witnessed or experienced violence, then coded respondents who experienced >2 events as experiencing community violence (1) *vs.* no exposure (0). Of note, only 13 youth (0.4% of our sample) had a parent report witnessing a violent death once but were otherwise coded as not being exposed to community violence because additional violent exposures were not reported. Cronbach’s alpha was 0.74.

#### Demographics

All analyses included youth age in months at the age 15 survey, youth-reported race/ethnicity at age 15 (non-Hispanic Black, Hispanic, non-Hispanic White, or non-Hispanic multiracial/other), and mother-reported youth sex at birth (male or female). To account for the unequal sampling of unmarried mothers at baseline, all analyses also included baseline maternal age, immigration status (U.S. born or immigrant), educational status (less than high school education, completed high school, some college or technical school, or a college degree), and relationship status with the youth’s biological father (married, cohabitating, or neither). Additionally, we controlled for household income at age 15 (categorized as <50% of the federal poverty level [FPL], 50–99% FPL, 100–199% FPL, 200–299% FPL, and ≥300% FPL).

### Analysis

We used Stata version 18.5 (StataCorp, 2025) for all analyses. Adjusted odds ratios and 95% confidence intervals are reported for all regression models.

#### Preliminary analysis

To contextualize our analytic sample and main findings within the existing literature base, we conducted preliminary logistic regressions to examine associations between youths’ cumulative ACE score (where the dichotomized ACE items were summed – ranging 0–11 – with higher scores indicating exposure to more ACE types) and each substance use outcome accounting for demographic covariates. We also conducted logistic regressions examining associations between individual ACEs and each substance use outcome accounting for demographic covariates, but not accounting for co-occurring ACEs.

#### Main analysis

For our main analysis, we evaluated descriptive statistics for our analytic sample, including the prevalence of substance use stratified by demographic characteristics and ACEs. Then, we conducted a series of logistic regressions to examine associations between individual ACEs and each substance use outcome accounting for demographic covariates and co-occurring ACEs (generating 3 models).

## Results

### Preliminary analysis

After accounting for demographic characteristics, cumulative ACE score was associated with cigarette use and marijuana use, but no association was observed for heavy alcohol use (Supplemental Table 1). After accounting for demographic characteristics but not co-occurring ACEs, neglect, parental problematic substance use, and parental incarceration were associated with cigarette use; emotional abuse was associated with heavy alcohol use; and emotional abuse, neglect, parental mental illness, parental incarceration, and parental IPV were associated with marijuana use (Supplemental Table 2).

### Main analysis

#### Descriptive statistics

Participants (*n* = 3200) were 14.4–18.6 years old, and 51.38% were male. The majority of participants identified as non-Hispanic Black (46.31%), followed by Hispanic/Latino (23.72%), and 30.22% were born into poverty. Prevalence of substance use was lower than expected based on 2015 national estimates (Kann et al., 2016); 12.89% of adolescents reported past-year marijuana use, 1.69% reported past-month cigarette use, and 2.6% reported past-year binge drinking. See Table 1 for additional information.

Prevalence of cigarette use was highest among youth who were male; 16.9–17.6 years-old; identified as multiracial or other race/ethnicity; had the lowest household incomes (<50% FPL); had mothers born outside the U.S.; and had mothers who, at baseline, were 21–26 years-old, had less than a high school education, and were cohabiting with the youth’s father. Prevalence of heavy alcohol use was highest among youth who were male; 17.8–18.6 years old; identified as non-Hispanic Black; had the highest household incomes (≥300% FPL); had mothers born outside the U.S.; and had mothers who, at baseline, were 33–38 years old, completed a high school education, and were married to the youth’s father. Prevalence of marijuana use was highest for youth who were male; 16.9–17.6 years old; identified as Hispanic/Latino; had the lowest household incomes (<50% FPL); had U.S.-born mothers; and had mothers who, at baseline, were 15–20 years old, had less than a high school education, and were neither married nor cohabiting with the youth’s father.

Table 2 describes the distribution of ACEs and substance use outcomes in our sample. Emotional abuse was the most prevalent ACE (80.88%) and approximately half the adolescents experienced physical abuse (52.16%). The least prevalent ACE was parental death (3.09%). When looking at ACEs overall, 19.39% of adolescents experienced one ACE, 31.36% experienced two ACEs, 21.75% experienced three ACEs, and 16.88% experienced four or more ACEs. The mean ACE score across the entire sample was 3.47 (SD: 1.89); adolescents reporting substance use were exposed to more ACEs than average. Notably, emotional and physical abuse were moderately correlated (*r* = 0.46), but other ACEs were weakly correlated with each other. See Supplemental Table 3 for additional information on ACE correlations.

#### Logistic regression results

After controlling for co-occurring ACEs, only emotional abuse was associated with higher odds of heavy alcohol use (aOR: 2.59 [1.23, 5.48]). Neglect and parental mental illness were associated with higher odds of marijuana use (aOR: 1.67 [1.08, 2.61] and 1.28 [1.01, 1.62], respectively). No significant associations were observed between individual ACEs and cigarette use. (Table 3)

## Discussion

This study investigated associations between 11 ACEs and adolescent marijuana, cigarette, and heavy alcohol use. Findings indicated specific ACEs may be particularly salient risk factors for distinct types of adolescent substance use. After accounting for co-occurring adversities, neglect, and parental mental illness were each associated with marijuana use. Emotional abuse (one of the most common ACEs reported nationally; CDC, 2024a, 2024b) was the primary ACE associated with heavy alcohol use. No individual ACEs emerged as significant predictors of cigarette use, after accounting for co-occurring adversities.

Current findings suggest well-documented associations between composite ACE indices and use of certain substances may be substantially driven by a narrow set of ACEs. While any exposure to violence during childhood is considered harmful and may disrupt children’s development, we proposed that some ACEs may be more strongly associated with specific outcomes (i.e., adolescent substance use) than other ACEs, with implications for ongoing ACE research and allocating scarce resources. Indeed, our findings that emotional abuse, and neglect and parental mental illness were prominent risk factors for heavy alcohol use and marijuana use, respectively, support this notion. Further, these findings somewhat align with existing, albeit limited, literature examining individual ACE effects on adolescent substance use (while controlling for co-occurring ACEs). Contrasting our findings, Afifi et al. (2020) reported that almost all individual ACEs in their study (which included emotional abuse, emotional neglect, household IPV, household substance misuse, household mental illness, parent separation/divorce, parental trouble with the police, spanking, poverty, community violence, parental gambling, and foster care/child protective services contact) were significantly associated with adolescent cigarette and marijuana use, as well as binge-drinking in a Canadian sample. However, similar to our findings, Karamanos et al. (2022) found a narrower set of ACEs were associated with general drug use in a United Kingdom sample, with slight variations noted at age 14 *vs.* age 17. Overall, they found gun violence, maternal problematic substance use, maternal IPV, and sexual assault were associated with drug use for males, and maternal problematic substance use, maternal IPV, sexual assault, and gun violence were associated with drug use for females.

Discrepancies between our results and those of earlier studies may be attributable to differences in sample characteristics or ACEs measured. Not only were the prior studies conducted with samples from other geographic regions, but they aimed to achieve representative samples (Afifi et al., 2020; Karamanos et al., 2022). This study examined a higher-risk sample. Further, Afifi et al. (2020) evaluated ACEs retrospectively (which may have introduced recall bias); whereas this study and Karamanos et al. (2022) evaluated ACEs prospectively during earlier childhood periods. Emerging literature also suggests that different ACEs may exert unique influences on outcomes dependent on the developmental timing of the ACE (e.g., physical abuse in early childhood *vs.* adolescence) (Bayer et al., 2006; Center for the Developing Child, 2007; Doom et al., 2022). However, this study and both existing studies did not account for the developmental timing of ACEs, which may have contributed to our discrepant findings. Lastly, the number and content of ACEs examined differed across this and the previous studies, with Afifi et al. (2020) including more ACEs than we did and Karamanos et al. (2022) including fewer ACEs. Due to the small number of existing studies and noted differences in study designs, results should be compared and interpreted cautiously, with continued research into these associations.

Notably, our findings partially align with emerging ACE research examining the distinct effects of ACE *subdomains* on various outcomes – comparing maltreatment-related ACEs to parent/household challenges-related ACEs. This literature largely reports that maltreatment-related ACEs are stronger predictors of poor outcomes than parent/household challenges-related ACEs (Fitzgerald & Gallus, 2025; Negriff, 2020; Sayyah et al., 2022). This aligns with our finding that emotional abuse was a singularly strong predictor of adolescent heavy alcohol use, and neglect was the strongest predictor of adolescent marijuana use. However, findings from studies that compare the maltreatment and parent/household challenges subdomains are not completely uniform (Garnsey et al., 2024; Sayyah et al., 2022; Wang et al., 2021). Considering our finding that parental mental illness was a significant predictor of adolescent marijuana use, while emotional and physical abuse were not significant predictors, it is possible that studies collapsing ACEs into subdomains may still be obscuring the importance of specific, individual ACEs for specific outcomes. Alternatively, recent research investigating ACE subdomains has highlighted that the developmental timing of maltreatment and parent/household challenges may be a possible confounder across this literature base (Bayer et al., 2006; Doom et al., 2022). Examining the effects of ACE timing during development was also outside the scope of this study but should be considered in future research.

When considering the current findings within the broader literature base, we also highlight results from our preliminary analyses which only partially align with existing evidence. We found cumulative ACEs were associated with adolescent cigarette and marijuana use, but not heavy alcohol use. The significant associations align with substantial existing literature reporting an association between cumulative ACEs and substance use broadly (Grummitt et al., 2022; Hoffmann & Jones, 2022). However, the null association between cumulative ACEs and heavy alcohol use contrasts existing literature (Hoffmann & Jones, 2022). Although similar to existing research (Gautam et al., 2023), we found adolescent heavy alcohol use was more prevalent for youth from higher-income households and our sample overrepresented socioeconomically disadvantaged youth. Thus, we hypothesize our null effects (and perhaps other findings) may have been influenced by our sample composition. Additionally, the models that evaluated individual ACEs separately (i.e., not accounting for co-occurring ACEs) produced multiple null associations, which was somewhat unexpected given substantial literature linking each of the ACEs with substance use (Hashim et al., 2025; McGovern et al., 2023; Whitesell et al., 2013). While we followed existing studies in the creation of our ACE measures, it is possible our operationalization of specific ACEs may have influenced our findings. However, it is also possible these differences may be attributable to underreporting of adolescent substance use or the unique characteristics of our sample.

Overall, our findings suggest researchers or practitioners that focus solely on the *number* of ACEs one experiences may be underestimating or overestimating individuals’ risk for certain negative outcomes by inappropriately assuming that all ACEs are equally weighted in their effects. This lends support to a growing concern within the ACEs field over limitations of the conventional approach to operationalizing ACEs as a count index (Fitzgerald & Gallus, 2025; McLennan et al., 2020), as well as concerns about ACE screening within clinical settings that cite ACE scores are not strong predictors of outcomes at the individual-level (Baldwin et al., 2021; McLennan et al., 2020). To be clear, we encourage practitioners to discuss ACEs, their potential negative sequelae, and potential supports with clients. Rather, we argue that conventional ACE screeners which indicate an adolescent with two ACEs – parental IPV and parental death – is at greater risk for heavy alcohol use compared to an adolescent with one ACE – emotional abuse – based on the ACE count alone is not accounting for the substantial association between emotional abuse and adolescent heavy alcohol use found in this study. Our findings suggest that understanding the specific ACEs to which an adolescent is exposed may be more informative for determining substance use risk than relying on ACE counts. However, there is more to investigate regarding the nuances of ACE exposures (e.g., severity, chronicity, perceived impact) relative to adolescent substance use (and other outcomes) which are beyond the scope of this study but may also be important for determining individuals’ risk for various outcomes. Ongoing research should consider using more-sophisticated analytic approaches to parse out the effects of individual and accumulating adversities. Lastly, the transition of several individual ACEs from significant to non-significant predictors once co-occurring ACEs were accounted for, suggests researchers examining the effects of individual ACEs should take a comprehensive approach to measuring and analyzing a wide range of ACEs simultaneously, so as to not incorrectly estimate the effects of any given ACE. For example, researchers investigating the association between emotional abuse and substance use, who do not account for physical abuse (which we found to be moderately correlated with emotional abuse in this study), may draw biased conclusions about the effects of emotional abuse alone based on the uncontrolled confounder.

### Limitations and strengths

This study has limitations. The sample is not nationally representative and consists of a single cohort, so generalizability is limited. Further, this adolescent data was from 2015. Contemporary adolescents are navigating and maturing within a different sociopolitical environment than our adolescent sample (e.g., having experienced the Covid-19 pandemic and its sequelae, experiencing major technological advancements). Additionally, data suggest decreasing trends in adolescent risk behaviors, including substance use, in recent years but worsening rates of depression and anxiety (CDC, 2024a, 2024b). Thus, findings from this study can provide some insight into risk factors for adolescent substance use but may not be directly generalizable to contemporary youth due to cohort effects. However, the sample includes many participants from subgroups traditionally underrepresented in research, including youth with minoritized ethnoracial identities and low socioeconomic status, which is a notable strength.

While we followed the convention of dichotomizing ACEs that is prevalent in ACEs literature, this approach loses potentially important information, like perceived impact and frequency/chronicity of exposures, which may provide greater insight into associations between ACEs and adolescent substance use. Additionally, the thresholds we used to dichotomize our ACEs may have masked effects associated with adversities less severe/persistent than those we included (Fitzgerald & Gallus, 2025; Mcguire et al., 2024). While alternative approaches to dichotomizing ACEs were possible, our approach aligns with existing literature (Gajos et al., 2023; Moore et al., 2025). Moreover, we decided to include youth whose parents participated in the age 3, 5, or 9 surveys (rather than requiring participation in all three waves) to maximize our sample size and ACE data. However, this approach means some youth had more opportunities for ACE identification than others. In other words, the 48% of youth who had parents participate in all three survey waves may have had more ACEs reported than the 25% of youth who only had a parent participate in one wave. Such underreporting may have influenced our findings. Future research should continue to explore the impact of how ACEs are operationalized in research.

Lastly, the prevalence of substance use within this sample was less than expected based on national estimates. Nationally-representative estimates of 10th graders in 2015 suggested 20% reported past-month marijuana use (*vs.* 13% of our sample who reported past-year use), 9% reported past-month cigarette use (*vs*. 2% of our sample), and 15% reported past-month heavy alcohol use (*vs*. 3% of our sample who reported past-year use) (Kann et al., 2016). These differences in prevalence may be due to social desirability bias and may have influenced our ability to detect associations between ACEs and our outcomes. Despite its limitations, this study is strengthened by its large sample size, prospective collection of ACE data throughout childhood, and timely, youth-reported substance use information.

## Conclusions

Our findings suggest emotional abuse may be a singularly salient risk factor for adolescent heavy alcohol use, and neglect and parental mental illness may be particularly strong risk factors for adolescent marijuana use. While any exposure to violence in childhood is considered harmful and may disrupt children’s development, our findings suggest particular attention should be paid to these ACEs when evaluating one’s risk for adolescent heavy alcohol and marijuana use. Continued research into these associations using alternative samples and a comprehensive set of ACEs is needed considering the limitations of existing literature and this study. Despite a need for continued research, current findings highlight that not all ACEs are equal relative to all outcomes, indicating future research examining ACE effects on health and wellbeing must critically consider how the operationalization of ACEs may influence findings. Further, practitioners using ACE counts as a means of estimating risk for adolescent substance use may significantly underestimate or overestimate risk. Findings also indicated that, when resources are limited, targeted strategies to decrease adolescent alcohol use should consider tailoring interventions for youth who have experienced emotional abuse, and strategies to decrease adolescent marijuana use should consider tailoring interventions to youth who have experienced neglect and/or parental mental illness to address those ACEs which were associated with greatest risk. Alternatively, findings from our preliminary and main analyses together suggest strategies aimed at decreasing adolescent cigarette use should consider tailoring interventions to youth who have experienced multiple ACEs, regardless of the specific ACEs experienced.

## Supplementary Material

Supp 1

Supplemental data for this article can be accessed online at https://doi.org/10.1080/10826084.2026.2679709.

## Figures and Tables

**Table 1.** Summary statistics of demographic characteristics and prevalence rates for substance use outcomes.

	Sample size (%)	Cigarette prevalence	Alcohol prevalence	Marijuana prevalence

Sample size				
Total sample	3200 (100)	–	–	–
Cigarette users	54 (1.69)	–	–	–
Heavy alcohol users	83 (2.60)	–	–	–
Marijuana users	411 (12.89)	–	–	–
Youth age, in years				
14.4–15.2	1382 (43.19)	0.87	1.30	8.32
15.3–16.0	1226 (38.31)	1.88	2.20	13.95
16.1–16.8	451 (14.09)	2.88	5.99	20.4
16.9–17.6	97 (3.03)	6.19	7.22	25.77
17.8–18.6	42 (1.31)	0.00	9.52	19.05
Youth Race/Ethnicity				
White	554 (17.31)	1.62	4.33	9.75
Black	1,482 (46.31)	1.55	1.21	13.36
Hispanic/Latino	759 (23.72)	2.11	3.82	13.97
Multiracial or other	238 (7.44)	2.52	4.20	13.87
Youth sex				
Male	1,644 (51.38)	2.19	2.62	14.48
Female	1,556 (48.62)	1.16	2.57	11.12
Maternal age, in years				
15–20	841 (26.28)	1.19	2.73	15.34
21–26	1,218 (38.06)	2.22	2.55	13.14
27–32	697 (21.78)	2.01	2.30	10.62
33–38	350 (10.94)	0.57	3.14	10.86
39–47	92 (2.88)	1.09	2.17	10.87
Maternal immigration status				
U.S. born	2751 (85.97)	1.67	2.40	13.34
Born outside U.S.	440 (13.75)	1.82	3.86	9.32
Maternal educational status				
Less than high school	990 (30.94)	2.22	2.63	16.67
Completed high school	1018 (31.81)	1.87	3.14	12.48
Some college/technical school	829 (25.91)	1.33	1.81	10.98
Completed college	359 (11.22)	0.56	2.79	7.52
Parental relationship status				
Married	790 (24.69)	1.39	3.16	8.35
Cohabiting	1149 (35.91)	1.83	2.87	14.1
Not married or cohabiting	1260 (39.38)	1.75	1.98	14.52
Household Income (FPL)				
<50%	432 (13.50)	2.78	2.08	18.29
50–99%	535 (16.72)	1.87	2.62	12.71
100–199%	909 (28.41)	1.76	2.64	12.98
200–299%	457 (14.28)	2.19	2.41	12.91
≥300%	851 (26.59)	0.71	2.94	10.11

*Note:* U.S.: United States; FPL: federal poverty level

Maternal characteristics were measured at baseline (i.e., the youth’s birth) and youth characteristics were measured at age 15.

**Table 2.** Summary statistics of individual and cumulative ACEs and prevalence rates for substance use outcomes.

	Sample size (%)	Cigarette prevalence	Marijuana prevalence	Alcohol prevalence	Mean total ACE score (SD)

Physical abuse					
No	1531 (47.84)	1.37	11.52	2.82	–
Yes	1669 (52.16)	1.98	14.16	2.40	–
Emotional abuse					
No	612 (19.12)	1.31	10.31	1.80	–
Yes	2588 (80.88)	1.78	13.5	2.79	–
Neglect					
No	3038 (94.94)	1.52	12.46	2.54	–
Yes	146 (4.56)	4.11	21.92	3.42	–
Parental problematic substance use					
No	2528 (79.0)	1.35	12.12	2.46	–
Yes	672 (21.0)	2.98	15.80	3.13	–
Parental mental illness					
No	1804 (56.38)	1.44	10.99	2.95	–
Yes	1396 (43.62)	2.01	15.34	2.15	–
Parental incarceration					
No	2444 (76.38)	1.35	11.38	2.42	–
Yes	756 (23.62)	2.78	17.77	3.18	–
Parental IPV					
No	2248 (70.25)	1.56	12.15	2.54	–
Yes	952 (29.75)	2.00	14.65	2.74	–
Parental death					
No	3101 (96.91)	1.68	12.82	2.62	–
Yes	99 (3.09)	2.02	15.15	2.02	–
Housing instability					
No	1288 (40.25)	1.09	10.35	2.57	–
Yes	1909 (59.66)	2.10	14.63	2.63	–
Food insecurity					
No	3019 (94.34)	1.72	12.67	2.62	–
Yes	181 (5.66)	1.10	16.67	2.21	–
Community violence					
No	2474 (77.31)	1.46	12.22	2.72	–
Yes	726 (22.69)	2.48	15.17	2.21	–
Mean total ACE score					
All individuals	3200 (100)	–	–	–	3.47 (1.89)
Cigarette users	54 (1.69)	–	–	–	4.35 (1.87)
Marijuana users	411 (12.89)	–	–	–	3.99 (1.90)
Heavy alcohol users	83 (2.60)	–	–	–	3.49 (1.85)

*Note*: IPV: intimate partner violence; SD: standard deviation

**Table 3.** Odds of adolescent substance use associated with youth’s individual ACE exposures after accounting for youth and maternal demographic covariates.

	Cigarette aOR [95% CI]	Alcohol aOR [95% CI]	Marijuana aOR [95% CI]

Physical abuse	1.38 [0.71, 2.67]	0.82 [0.50, 1.35]	1.07 [0.83, 1.37]
Emotional abuse	1.19 [0.47, 3.02]	2.59 [1.23, 5.48]*	1.25 [0.88, 1.76]
Neglect	1.98 [0.79, 4.98]	1.43 [0.55, 3.72]	1.67 [1.08, 2.61]*
Parental problematic substance use	1.53 [0.81, 2.89]	0.95 [0.53, 1.71]	0.99 [0.75, 1.30]
Parental mental illness	1.06 [0.58, 1.93]	0.66 [0.40, 1.09]	1.28 [1.01, 1.62]*
Parental incarceration	1.63 [0.87, 3.05]	1.65 [0.92, 2.94]	1.28 [0.99, 1.67]
Parental IPV	1.13 [0.61, 2.07]	1.1 [0.66, 1.82]	1.15 [0.90, 1.47]
Parental death	1.51 [0.35, 6.52]	1.26 [0.29, 5.39]	1.31 [0.73, 2.35]
Housing instability	1.24 [0.63, 2.45]	1.14 [0.67, 1.94]	1.1 [0.85, 1.42]
Food insecurity	0.37 [0.08, 1.59]	1.03 [0.36, 3.01]	0.93 [0.58, 1.49]
Community violence	1.47 [0.78, 2.77]	0.94 [0.50, 1.75]	0.98 [0.75, 1.28]

*Note*: aOR: adjusted odds ratio; CI: confidence interval; IPV: intimate partner violence

* Indicates significance at *p* < 0.05. A total of 3 models are represented here. All models controlled for youth age, sex, and race/ethnicity, as well as maternal baseline age, immigration status, educational status, relationship status with youth’s biological father, and household income (relative to needs).

## Data Availability

We use publicly available data that can be accessed upon application from the Future of Families and Child Wellbeing Study (FFCWS) website (https://ffcws.princeton.edu/).
