# Supplementary material for: Individual Contributions of Adverse Childhood Experiences to Adolescent Substance Use
Source: Subst Use Misuse. Author manuscript; Available in PMC 2026 Jul 10. (PMC13352494; doi:10.1080/10826084.2026.2679709)
Supplement: Supp 1 [file NIHMS2185035-supplement-Supp_1.pdf]

Supplemental Materials

Supplemental Table 1. Odds of adolescent substance use associated with youth's cumulative ACE score.

	**Cigarette OR [95% CI]**	**Alcohol OR [95% CI]**	**Marijuana OR [95% CI]**
**ACE Score**	1.25 [1.08, 1.45]*	1.08 [0.95, 1.23]	1.14 [1.07, 1.21]*
**Youth Age**	1.06 [1.03, 1.08]*	1.07 [1.05, 1.10]*	1.05 [1.04, 1.07]*
**Youth Race/Ethnicity**			
White	(ref)	(ref)	(ref)
Black	0.54 [0.23, 1.26]	0.21 [0.11, 0.43]*	0.95 [0.66, 1.36]
Hispanic/Latino	0.83 [0.33, 2.09]	0.64 [0.33, 1.26]	1.3 [0.87, 1.93]
Multiracial or other	1.17 [0.4, 3.45]	0.84 [0.38, 1.85]	1.38 [0.85, 2.25]
**Youth Sex**			
Male	(ref)	(ref)	(ref)
Female	0.53 [0.29, 0.94]*	0.97 [0.62, 1.53]	0.75 [0.60, 0.94]*
**Maternal Age**	1.00 [0.95, 1.06]	0.99 [0.94, 1.03]	1.0 [0.98, 1.02]
**Maternal Immigration Status**			
U.S. born	(ref)	(ref)	(ref)
Born outside U.S.	1.05 [0.43, 2.57]	1.26 [0.65, 2.46]	0.61 [0.41, 0.92]*
**Maternal Educational Status**			
Less than high school	(ref)	(ref)	(ref)
Completed high school	1.02 [0.53, 1.97]	1.38 [0.77, 2.45]	0.78 [0.59, 1.02]
Some college or technical school	0.81 [0.37, 1.81]	0.78 [0.38, 1.61]	0.77 [0.56, 1.06]
College degree or more	0.48 [0.09, 2.64]	0.83 [0.30, 2.29]	0.76 [0.43, 1.32]
**Parental Relationship Status**			
Married	(ref)	(ref)	(ref)
Cohabiting	0.75 [0.33, 1.70]	0.85 [0.45, 1.62]	1.33 [0.93, 1.91]
Not Married or cohabiting	0.75 [0.32, 1.76]	0.78 [0.39, 1.58]	1.37 [0.95, 1.99]
**Household Income (FPL)**			
<50%	1.56 [0.71, 3.45]	1.05 [0.47, 2.37]	1.33 [0.95, 1.86]
50-99%	1.09 [0.48, 2.45]	1.16 [0.58, 2.33]	0.97 [0.69, 1.36]
100-199%	(ref)	(ref)	(ref)
200-299%	1.39 [0.62, 3.15]	0.92 [0.42, 2.00]	1.06 [0.74, 1.51]
≥300%	0.53 [0.19, 1.48]	1.28 [0.65, 2.54]	1.11 [0.79, 1.56]

Note: Abbreviations are ACE, adverse childhood experience; OR, odds ratio, CI, confidence interval, U.S., United States; FPL, federal poverty level, (ref), reference category. * indicates significance at p<0.05.
Maternal characteristics were assessed at the baseline survey (i.e. the focal youth's birth).

Supplemental Table 2. Odds of adolescent substance use associated with youth's individual ACE exposures after accounting for youth and maternal demographic covariates but not co-occurring ACEs.

	Cigarette<br>aOR [95% CI]	Alcohol<br>aOR [95% CI]	Marijuana<br>aOR [95% CI]
**Physical Abuse**	1.48 [0.83, 2.61]	1.07 [0.67, 1.70]	1.20 [0.96, 1.50]
**Emotional Abuse**	1.55 [0.71, 3.38]	2.43 [1.21, 4.88]*	1.38 [1.02, 1.88]*
**Neglect**	2.55 [1.05, 6.18]*	1.65 [0.64, 4.24]	1.89 [1.22, 2.91]**
**Parental Problematic Substance Use**	1.94 [1.08, 3.48]*	1.13 [0.66, 1.95]	1.16 [0.90, 1.50]
**Parental Mental Illness**	1.21 [0.69, 2.10]	0.73 [0.46, 1.18]	1.37 [1.10, 1.71]**
**Parental Incarceration**	1.86 [1.02, 3.39]*	1.53 [0.88, 2.66]	1.36 [1.06, 1.75]*
**Parental IPV**	1.28 [0.72, 2.28]	1.11 [0.68, 1.83]	1.27 [1.01, 1.61]*
**Parental Death**	1.31 [0.31, 5.58]	1.07 [0.25, 4.53]	1.26 [0.70, 2.24]
**Housing Instability**	1.67 [0.88, 3.20]	1.19 [0.72, 1.95]	1.25 [0.98, 1.60]
**Food Insecurity**	0.51 [0.12, 2.14]	0.97 [0.34, 2.76]	1.07 [0.68, 1.69]
**Community Violence**	1.56 [0.85, 2.87]	0.99 [0.54, 1.81]	1.08 [0.84, 1.41]

Note: Abbreviations are ACE, adverse childhood experience; aOR, adjusted odds ratio; CI, confidence interval; IPV, intimate partner violence. * indicates significance at $p<0.05$ and **$p<0.01$.
A total of 33 models are represented here – one model for each outcome and each ACE. All models controlled for youth age, sex, and race/ethnicity, as well as maternal baseline age, immigration status, educational status, relationship status with youth's biological father, and household income (relative to needs).

Supplemental Table 3. Correlations of the adverse childhood experience (ACE) variables.

	Physical Abuse	Emotional Abuse	Neglect	Parent Problematic Substance Use	Parent Mental Illness	Parent Incarceration
**Emotional Abuse**	0.46					
**Neglect**	0.07	0.07				
**Parent Problematic Substance Use**	0.06	0.06	0.08			
**Parent Mental Illness**	0.09	0.1	0.08	0.17		
**Parent Incarceration**	0.08	0.06	0.09	0.17	0.17	
**Parent Intimate Partner Violence**	0.07	0.06	0.07	0.09	0.17	0.08
